# miRTargetLink—miRNAs, Genes and Interaction Networks

**DOI:** 10.3390/ijms17040564

**Published:** 2016-04-14

**Authors:** Maarten Hamberg, Christina Backes, Tobias Fehlmann, Martin Hart, Benjamin Meder, Eckart Meese, Andreas Keller

**Affiliations:** 1 Chair for Clinical Bioinformatics, Saarland University, Saarbrücken D-66041, Germany; e.j.m.hamberg@gmail.com (M.Ham.); c.backes@mx.uni-saarland.de (C.B.); tobias.fehlmann@ccb.uni-saarland.de (T.F.); 2 Department of Human Genetics, Saarland University, Homburg D-66421, Germany; marhar15@googlemail.com (M.Har.); hgemee@uks.eu (E.M.); 3 Internal Medicine, Heidelberg University, Heidelberg D-69120, Germany; benjamin.meder@med.uni-heidelberg.de

**Keywords:** miRTargetkLink, miRNAs, genes, interaction networks

## Abstract

Information on miRNA targeting genes is growing rapidly. For high-throughput experiments, but also for targeted analyses of few genes or miRNAs, easy analysis with concise representation of results facilitates the work of life scientists. We developed miRTargetLink, a tool for automating respective analysis procedures that are frequently applied. Input of the web-based solution is either a single gene or single miRNA, but also sets of genes or miRNAs, can be entered. Validated and predicted targets are extracted from databases and an interaction network is presented. Users can select whether predicted targets, experimentally validated targets with strong or weak evidence, or combinations of those are considered. Central genes or miRNAs are highlighted and users can navigate through the network interactively. To discover the most relevant biochemical processes influenced by the target network, gene set analysis and miRNA set analysis are integrated. As a showcase for miRTargetLink, we analyze targets of five cardiac miRNAs. miRTargetLink is freely available without restrictions at www.ccb.uni-saarland.de/mirtargetlink.

## 1. Introduction

Biochemical networks play a central role in life science research. Tools for visualizing and analyzing such networks have a long history. Among the most popular web-based applications is STRING. Originally implemented as a search tool for recurring instances of neighboring genes [1], STRING v10 has become a comprehensive protein-protein interaction database containing predicted and validated protein-protein target information [2]. The success of this web service and database is not only driven by the large amount of information in the database but also the intuitive manner in its running analyses. Entering one or several protein identifiers enables comprehensive analysis of protein-protein interactions and deriving important information on a network level.

With non-coding RNAs gaining importance in biomedical research, similar tools with respect to miRNA-gene interactions have been presented. We here exemplarily mention three web services that involve the analysis of miRNAs and target genes. Among the first tools, we published miRTrail [3]. Tailored for high-throughput omics analyses, deregulated genes, miRNAs and target information can be uploaded to find clusters. Hamed and co-workers presented TFmiR, a web service for constructing and analyzing transcription factor and miRNA co-regulatory networks in a disease-specific manner [4]. One further tool in a similar direction is TargetCompare, a web interface for studying multiple targets of pre-selected miRNAs [5].

Generally, web services of miRNA-gene interactions require file data upload (e.g., gene or miRNA expression) and are tailored for specific applications. The majority of available tools are designated predominantly for large-scale experimental data such as microarrays and high-throughput sequencing. Many researchers work, however, with single genes or miRNAs or, at most, with small sets. If regulatory information is included in respective research projects, targets are frequently extracted manually from databases and small networks are drawn manually or using tools such as Cytoscape [6]. Taking STRING as an archetype, we implemented miRTargetLink. Users can enter single miRNAs, genes or sets of miRNAs and genes (ranging from two or three up to very comprehensive sets from high-throughput experiments) in the web interface. Interaction networks based on the information from miRTarBase [7] and miRanda [8] are calculated and visualized. Users can modify the resulting network, e.g., only validated targets with strong evidence can be considered. For downstream analysis we have built an interface to GeneTrail2, an updated version of the GeneTrail web service [9].

In this manuscript we describe miRTargetLink, as well as its input, output and the functionality. We also mention current limitations and forward research directions. On the webpage of miRTargetLink [10], example input is provided and alongside other relevant information, a detailed tutorial is also available.

## 2. Results

One of the major goals of miRTargetLink, besides functionality, was a user-friendly interface. To provide a convenient and standardized solution that runs on state-of-the art web browsers, miRTargetLink is implemented in php 5 and JavaScript. Interactions are stored in a MySQl database that is regularly updated. The interaction networks are generated with the networks visualization package from the VIS.js JavaScript library version 4 [11].

## 3. Data Input

An important feature of miRTargetLink is the straightforward data input. Single miRNA identifiers, gene identifiers or sets of miRNAs and genes can be copy-pasted in the web interface. No file data upload is required. For gene identifiers, the gene symbol is used, and miRNAs are uploaded following the current nomenclature that is also used by miRBase [12]. Since many research projects have been carried out on information from older miRBase versions (using the * annotation for minor forms of miRNAs), we implemented a web-based mapping tool that converts identifiers from older versions to the most recent annotation [13]. The converted IDs can then be provided to miRTargetLink. Gene and miRNA identifiers are matched automatically; if several hits are found for a provided ID, the user can select the right hit. If, for example, “let-7a” is entered, the three matching hits hsa-let-7a-2-3p, hsa-let-7a-3p and hsa-let-7a-5p are proposed by miRTargetLink. In the single gene/miRNA mode, the correct ID is selected by a radio button. In the multi-miRNA/gene mode, for each gene/miRNA the number of hits is shown and the correct ID can be selected from a drop-down list.

## 4. Constructing, Visualizing and Modifying Networks

From the input, target information is extracted from miRTarBase [7], one of the most comprehensive miRNA-gene target resources presently available. Predicted targets are added based on the miRanda algorithm. From the data, an interaction network is calculated and visualized.

**Figure 1:**
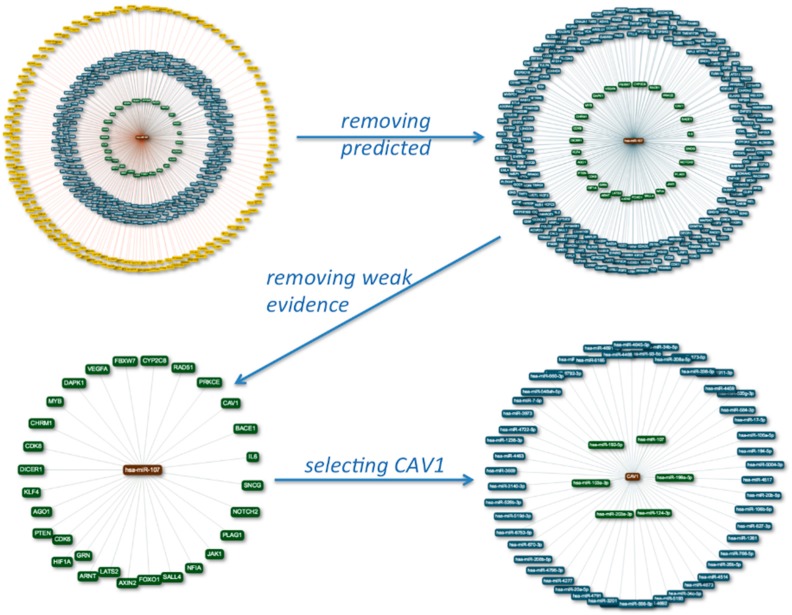
Single miRNA mode for miR-107: The central node is the miRNA, surrounded by validated targets with strong evidence (green), weak evidence (blue), and predicted targets (yellow). In the second representation predicted edges and in the third weak evidence edges are removed. The final representation is gene-centric for one of the target gens of miR-107, *CAV1*.

As an example, for the single miRNA mode the network for miR-107 is presented in Figure 1, where the central brown node is the miRNA, the green nodes in the inner circle are validated targets with strong evidence (e.g., luciferase assay), the blue genes in the middle circle are validated by weak evidence (e.g., microarray), and the yellow nodes in the outer circle are the predicted targets. The second image is the result of removing the predicted targets. The representation in the lower left part results from removing weak experimental evidence targets as well. By double-clicking a gene node, e.g., *CAV1*, the network-centric view for this gene is generated (lower right part). Now the miRNAs that are known to or predicted to target *CAV1* are presented in the respective colors. In each case, the current interactions of the network are dynamically listed in tabular form below the visualization widget. The respective table can also be downloaded as a text file. The genes and miRNAs in that table are linked to GeneCards and miRBase. The network visualization can be saved as a png file using the right mouse button.

**Figure 2:**
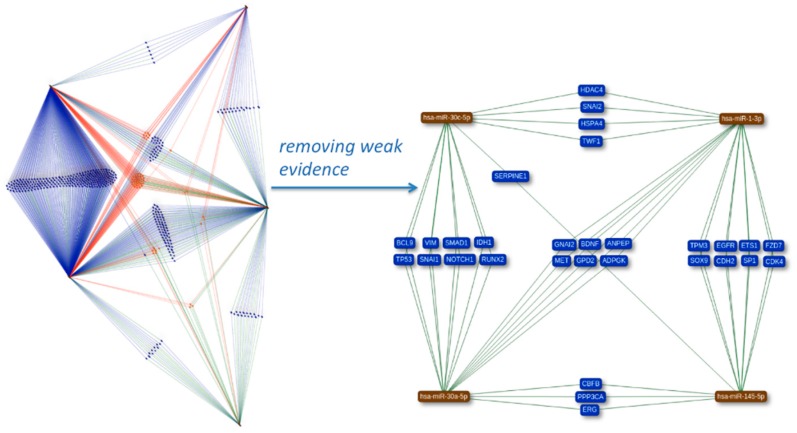
Multi-miRNA mode. For five cardio miRNAs, the network of genes that are targeted by at least two miRNAs (experimental evidence) is shown. Edge color indicates the number of miRNA-target interactions for the miRNAs that are start nodes of the edges. The second representation highlights the genes that are supported by strong evidence experiments.

Often, researchers are, however, not only interested in single genes or miRNAs but in sets of the respective molecules. Thus, miRTargetLink can also handle comma-separated lists of identifiers, both for genes and miRNAs. As a case study, we used five known cardio-miRNAs as input, including hsa-miR-1-3p [14,15], hsa-miR-145-5p [16,17], hsa-miR-30a-5p [17], hsa-miR-30c-5p [17], hsa-miR-423-5p [15]. Users can select the interactions to be included in the network, *i.e.*, weak or strong evidence-validated interactions. The network containing all validated targets for the five cardio miRNAs is presented in Figure 2. The color of the genes indicates the number of interactions. Orange genes are targeted by three or more microRNAs, genes that are targeted by two microRNAs are blue. Interactions with strong experimental evidence are depicted by green edges while interactions with weaker evidence are depicted by blue (genes with two edges) or orange (genes with more than two edges). As for the single gene/miRNA mode, weak evidence edges can be removed. The resulting network for the same miRNAs is presented in the right part of Figure 2. Genes or miRNAs can be marked from this network and novel sub-networks can be generated from the marked nodes in a new window, making the multi-gene/miRNA mode as interactive as the single-gene mode.

## 5. Enrichment Analysis

As result of the target analysis, sets of miRNAs and genes are generated dynamically. An important aspect for researchers is the enrichment of genes or miRNAs in biochemically relevant categories such as pathways. We implemented an interface to GeneTrail2 [18], which builds up on the GeneTrail framework [9]. GeneTrail2 provides the respective functionality for systems biological analysis of the sets and enables over-representation analysis with a single mouse click. As a result, gene ontology categories, KEGG pathways, transcription factors or genomic clusters that are enriched for genes in the network are presented. Although single miRNAs can potentially regulate pathways [19], this analysis functionality is tailored for sets of miRNAs, e.g., in the case of the five cardio miRNAs, we find enrichment for cancer-related miRNAs, driven by markers that are enriched in the cell cycle and the apoptosis. Among the most significant KEGG pathways we observe “Arrhythmogenic right ventricular cardiomyopathy” (Benjamini-Hochberg adjusted *p*-value of 1.6 × 10^−4^) with genes *JUP*, *ACTB*, *ITGA6*, *DSG2*, *ATP2A2*, *ITGB4*, *DAG1* and *CDH2*. Also “Dilated cardiomyopathy” with genes *ACTB*, *ITGA6*, *ATP2A2*, *ITGB4*, *DAG1* and *TPM3* remained significant (Benjamini-Hochberg adjusted *p*-value of 6.6 × 10^−3^).

## 6. Limitations and Future Research Directions

We consider miRTargetLink as an ongoing research project. Thus, we want to mention current limitations of the tool and three ongoing research efforts.

(1) At this stage, the application is limited to *Homo sapiens* since most information is available and a considerable amount of biomedical research is carried out in humans. In the next release we will integrate other organisms, starting with *Mus musculus* and *Rattus norvegicus*. Along with more organisms, improved mapping functionality and support of other identifiers can facilitate the application of miRTargetLink.
(2) Validated miRNA gene interactions are central for miRTargetLink. As a comprehensive resource for such interactions, we selected the miRTarBase. In addition to this database, many others are available (e.g., Tarbase or miRecords). To extend the tool beyond validated targets, predicted interactions are included. However, respective targets are known to depend on the prediction algorithm. Results can vary tremendously between different algorithms. In the present release, predicted interactions in miRTargetLink rely on miRanda. We have selected miRanda as a case of frequently applied prediction algorithms. Adding other prediction tools can potentially improve the results provided by miRTargetLink. Before adding those, the performance of the different methods, however, has to be evaluated critically to validate interactions.
(3) The miRNA-gene predictions are known to be error-prone. This means that some of the miRNA-gene interactions that have been validated by life scientists may have been negative. The negative experiments are usually not reported. We are implementing a database for negative miRNA-gene interactions and will integrate the information in miRTargetLink so that potential users also get the information on false positive predictions.

## 7. Discussion

With miRTargetLink we present a web interface facilitating low- and medium-throughput analysis of miRNAs and target genes. We mainly address applied life scientist researchers who work with single genes, miRNAs or small sets to facilitate tasks that are currently done on a daily basis but in a semi-automated fashion.

While miRTargetLink is not thought to be a replacement for existing high-throughput miRNA analytics tools that also include quantitative analysis (such as miRTrail [3], TFmiR [4] or TargetCompare [5]), our program can also be used for quantitative high-throughput miRNA and gene expression analyses, e.g., from microarrays or Next Generation Sequencing, we implemented straightforward data input and handling. Additional information along with a detailed tutorial is provided online. Currently implemented for *Homo sapiens*, we consider miRTargetLink an ongoing research project. Extension to other model organisms is a reasonable next step, together with additional background information such as false positive miRNA-gene interactions that have been proven to be wrong in functional experiments.
